# The genera *Areopraon* Mackauer, 1959 and *Pseudopraon* Starý, 1975 (Hymenoptera, Braconidae, Aphidiinae) from China, with keys to species

**DOI:** 10.3897/zookeys.780.26264

**Published:** 2018-08-08

**Authors:** Hong-Wei Tian, Cornelis van Achterberg, Xue-Xin Chen

**Affiliations:** 1 State Key Laboratory of Rice Biology and Ministry of Agriculture Key Lab of Molecular Biology of Crop Pathogens and Insect Pests, Institute of Insect Sciences, Zhejiang University, Hangzhou 310058, China Zhejiang University Hangzhou China; 2 Key Laboratory of Resource Biology and Biotechnology in Western China (Northwest University) and Ministry of Education, College of Life Sciences, Northwest University, 229 North Taibai Road, Xi’an, Shaanxi 710069, China Northwest University Xi'an China

**Keywords:** parasitoid wasp, Aphidiinae, *
Areopraon
*, China, key, new species, Oriental, Praini, *
Pseudopraon
*, Yunnan, Zhejiang

## Abstract

Two genera, *Areopraon* Mackauer, 1959 and *Pseudopraon* Starý, 1975, are newly recorded from China in this paper. Two new species, namely *A.chui* Tian & Chen, **sp. n.** and *P.hei* Tian & Chen, **sp. n.**, are described and illustrated. Keys to the known species of these two genera are provided.

## Introduction

The genus *Areopraon* was erected by Mackauer in 1959 with eight known species at present (Davidian and Gavrilyuk 2011). The species of *Areopraon* have two types of pupation behavior simultaneously: internal (within the host aphid) and external (under the host aphid) (Starý 1976; Tobias and Kyriac 1971). They usually attack aphids of the families Aphididae (mainly the subfamilies Pterocommatinae, Pemphiginae, and Chaitophorinae) and Eriosomatidae that usually produce a waxy cover and galls on their host plants (Mackauer 1959; Starý 1976).

The genus *Pseudopraon* was erected by Starý in 1975 based on specimens reared from *Mindarusabietinus* Koch, 1857 (Homoptera: Mindaridae) on *Abiesalba* in the Czech Republic (Starý 1975), and used to be treated as a monotypic genus (Tobias and Kyriac 1971; Starý 1975; Tomanović et al. 2006; Tomanović et al. 2009). The type species, *P.mindariphagum* Starý, 1975, also has two types of pupation behavior simultaneously (Starý 1975).

*Pseudopraon* is a sister group to *Areopraon* (Tomanović et al. 2006), and can be clearly separated by having an entirely smooth propodeum (usually having areola or carina in *Areopraon*), and the absence of vein 1-SR+M of fore wing (at least partly present in *Areopraon*).

Both *Areopraon* and *Pseudopraon* are here newly recorded from China, and two new species, *A.chui* Tian & Chen, sp. n. and *P.hei* Tian & Chen, sp. n. are described and illustrated. Identification keys to the known species of these two genera are also provided.

## Materials and methods

Specimens studied are deposited in the Parasitic Hymenoptera Collection of Zhejiang University, Hangzhou, China (**ZJUH**). Descriptions and measurements were made under a stereomicroscope (Zeiss Stemi 2000). All photographs were made by a digital camera (KEYENCE VHX-2000C) with a KEYENCE VH-Z20R lens and processed with Adobe Photoshop CS5.0, mostly to adjust the size and background. Terminology follows van Achterberg (1988), veins follow the modified Comstock-Needham system (van Achterberg, 1979).

Abbreviations used in this paper are as follows:

**POL** distance between hind ocelli

**Od** maximum diameter of hind ocellus

**T1** first tergite of the metasoma

**F1** first flagellomere of the antenna (or third antennal segment)

**F2** second flagellomere of antenna

## Taxonomy

### Areopraon

Taxon classificationAnimaliaHymenopteraBraconidae

Mackauer, 1959

Areopraon Mackauer, 1959: 810. Type-species: Praonlepelleyi Waterston, 1926.
Mesopraon Starý, 1981: 175. (Syn. by Tomanović, Ž.). Type-species: Mesopraonhelleni Starý, 1981.

#### Diagnosis.

Head transverse, with occipital carina. Maxillary palpi with four segments, labial palpi with three segments. Female antenna filiform, with 12–22 segments. Notauli deep and distinct throughout. Propodeum usually with areola or with carinae. Fore wing with pterostigma longer than vein 1-R1 (= metacarp), radial vein (= r+3-SR) not reach the wing apex, vein 1-SR+M usually faintly indicated, and vein 2-M always distinct. Hind wing with cross vein cu-a absent. Apex of ovipositor sheaths usually densely pubescent. Larva pupates either inside or outside of mummified aphid.

##### Key to world species of the genus *Areopraon* Mackauer, 1959

**Table:** 

1	Fore wings without vein m-cu (= recurrent vein), or faintly indicated; carinae or areola of propodeum not always present	**2**
–	Vein m-cu of fore wing distinctly present (Figure 2A); propodeum with distinct carinae or areola (Figure 2J)	**4**
2	Propodeum with distinct carinae posteriorly; mesoscutum almost hairless; antenna of female with 12 segments	***A.thailandicum* Starý, 2008**
–	Propodeum without any carinae; mesoscutum densely pubescent; antenna with more than 12 segments	**3**
3	Antenna of ♂ with 19–20 segments; pterostigma less slender, no more than 3.0× as long as wide	***A.antiquum* Mackauer, 1967**
–	Antenna of ♀ with 15 segments; pterostigma slender, 5.0× as long as wide	***A.rasnitsyni* Davidian, 2011**
4	Propodeum with complete areola (Figure 2J)	**5**
–	Propodeum without complete areola, only with distinct carinae posteriorly	**8**
5	T1 slender, at least 1.6× as long as wide at spiracle level; antenna of ♀ with 17–18 segments	**6**
–	T1 less slender or nearly subquadrate; Antenna of ♀ with 19–20 segments	**7**
6	Mesoscutum glabrous, with medial and lateral lobes nearly glabrous, with any setae (Fig. 2C, D); T1 2.3× as long as wide at spiracle level (Figure 2F); Pterostigma triangular, 3.4× as long as wide (Figure 2A); Antenna of ♀ with 18 segments (Figure 2I)	***A.chui* Tian & Chen, sp. n.**
–	Mesoscutum densely pubescent, only with small hairless area; T1 1.6× as long as wide at spiracle level; Pterostigma triangular, 4.0× as long as wide; Antenna of ♀ with 17 segments	***A.helleni* (Starý, 1981)**
7	Antenna of ♀ with 19–20 segments; T1 1.2–1.5× as long as wide at spiracle level	***A.silvestre* (Starý, 1971)**
–	Antenna of ♀ with 22 segments; T1 subquadrate, nearly as long as wide at spiracle level	***A.pilosum* Mackauer, 1959**
8	Antenna of ♀ with 13–14 segments; T1 subquadrate, nearly as long as wide at spiracle level	***A.lepelleyi* (Waterston, 1926)**
–	Antenna of ♀ with 16–17 segments; T1 1.25–1.33× as long as wide at spiracle level	***A.chaitophori* Tomanović & Petrović, 2009**

### Areopraon chui

Taxon classificationAnimaliaHymenopteraBraconidae

Tian & Chen sp. n.

http://zoobank.org/EF57B900-B6C6-4D41-A6B1-F78BA9055A46

Figures 1 , 2

#### Description.

Female. Body length 2.6 mm, fore wing length 2.2 mm.

*Head*. Head transverse in dorsal view, slightly wider than mesoscutum, smooth and shiny, with sparse long setae (Fig. 2H). Eyes medium-sized (Figure 2G), oval, sparsely setose. Temple in dorsal view 1.4 times as long as eye. Malar space equal to 0.15× longitudinal diameter of eye. POL 1.0× Od. Width of face 1.4× its height and 0.4× width of head. Face with several setae. Clypeus oval, raised with several long setae, tentoriocular line equal to 0.3 of inter-tentorial line. Antenna filiform, with 18 segments. F1 approx. 1.3 times longer than F2. F1 4.3× as long as wide, F2 2.5× as long as wide (Figure 2I).

**Figure 1.**
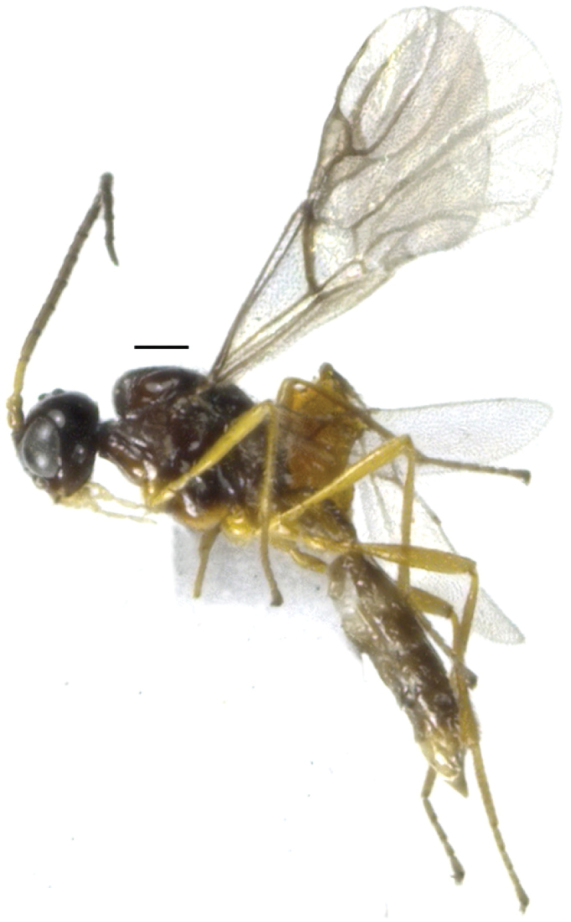
*Areopraonchui* Tian & Chen, sp. n. Habitus, lateral aspect. Scale bar: 0.2 mm.

**Figure 2.**
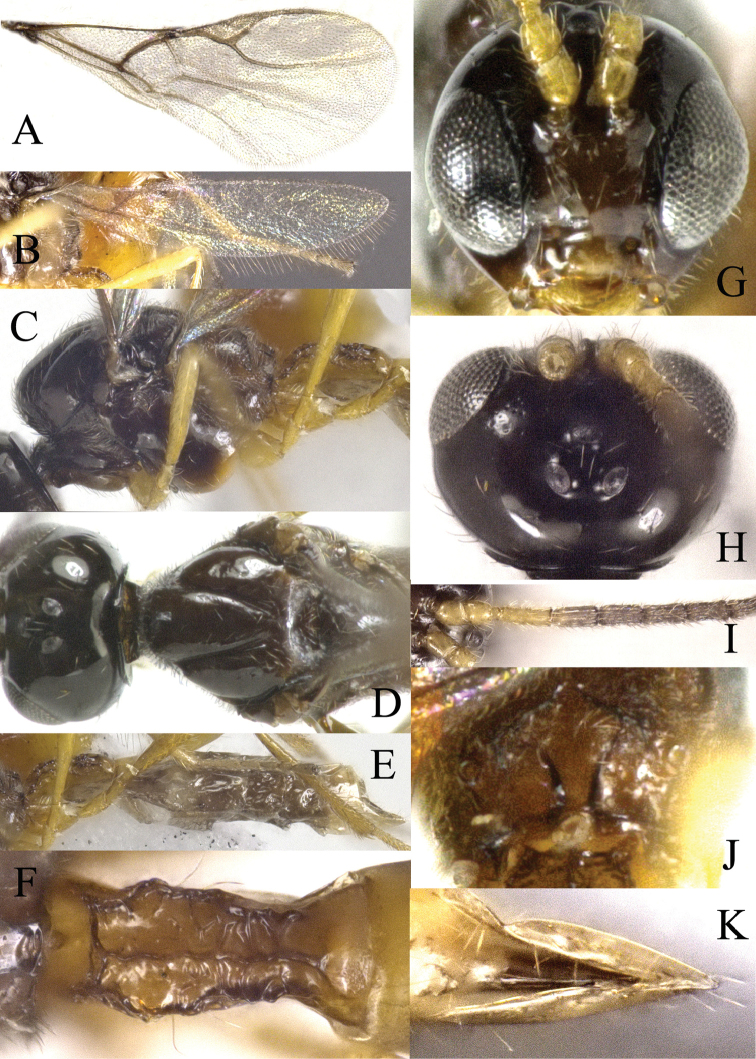
*Areopraonchui* Tian & Chen, sp. n. **A** fore wing **B** hind wing **C** mesosoma, lateral aspect **D** mesoscutum, dorsal aspect **E** metasoma, lateral aspect **F**T1, dorsal aspect **G** head, anterior aspect **H** head, dorsal view **I** antenna **J** propodeum, dorsal aspect **K** ovipositor sheaths, dorsal aspect.

*Mesosoma*. Mesonotum with central and lateral lobes glabrous, covered with several long setae. Notauli deep and distinct throughout (Figure 2D). Propodeum with complete areola (Figure 2J).

*Wings*. Apical margin of fore wing with long setae, which are longer than setae on wing membrane. Pterostigma triangular, 3.4× as long as wide. Distal abscissa of 1-R1 (= metacarp) 0.7× as long as pterostigma. Radial vein (= r+3-SR) 1.5× as long as 1-R1, do not reach the wing apex. Basal half of SR+M vein distinctly pigmented (Figure 2A).

*Metasoma*. T1 with medial and lateral carinae, 2.3× as long as wide at spiracle level, with two long setae each side close to spiracle (Figure 2F). Ovipositor sheaths glabrous, except the apex (Figure 2K).

*Colour*. Head dark brown, face somewhat paler, clypeus and mandibles yellowish to light brown. Maxillary and labial palpi white to yellowish. Antennal scape, pedicel and F1 yellowish to brown. The ventral view of mesosoma and propodeum brown. Wings hyaline with brown venation. Legs yellowish to light brown, apices of tarsi dark. Remainder of antenna and mesosoma dark brown. Metasoma and ovipositor sheath brown.

**Male.** Unknown.

#### Host.

Unknown.

#### Material examined.

Holotype: ♀, S China, Zhejiang, Mt. Qingliang, 16.V.2012, Tang Pu, No.201205480.

#### Distribution.

China (Zhejiang).

#### Taxonomic remarks.

This species is similar to *Areopraonhelleni* Starý, 1981, by having T1 very long, but can be separated by the differences listed in the above key. It is also similar to *A.thailandicum* Starý, 2008, by having the mesoscutum nearly hairless, but can be distinguished by having the vein m-cu of fore wing distinctly present (the latter completely absent) and the antenna 18-segmented (the latter 12-segmented).

#### Etymology.

The species is named in honour of Prof. Chu Joo-tso (ZJUH), the well-known Chinese hymenopterist.

### Pseudopraon

Taxon classificationAnimaliaHymenopteraBraconidae

Starý, 1975

Pseudopraon Starý, 1975: 249. Type species: Pseudopraonmindariphagum Starý, 1975.

#### Diagnosis.

Head transverse. Eyes small. Maxillary palpi 4-segmented, labial palpi 2-segmented. Antenna filiform, with the number of segments different in both sexes. Mesoscutum with the notauli completely developed. Propodeum smooth. Fore wing with vein 1-R1 (= metacarp) intermediate in length; vein r+3-SR (= radial vein) partially distinct, feebly indicated up to the wing apex; vein 1-SR+M absent; and vein m-cu+2-M feebly pigmented but distinctly present. Hind wing with basal cell complete. Metasoma lanceolate in the female, robust at apex in the male. T1 quadrate. Ovipositor lanceolate at the apex. Ovipositor sheaths narrowed to the apex, slightly arcuate, shortly pubescent.

##### Key to world species of the genus *Pseudopraon* Starý, 1975

**Table:** 

1	Ovipositor sheaths densely pubescent; Vein 1-R1 (= metacarp) half the length of pterostigma; T1 subquadrate, slightly longer than wide at spiracle level (10:9); antenna of female with 12–13 segments	***P.mindariphagum* Starý, 1975**
–	Ovipositor sheaths less pubescent (Figure 3J); Vein 1-R1 0.7× the length of pterostigma (Figure 3G); T1 1.2–1.3× as long as wide at spiracle level (Figure 3E); antenna of female with 18 segments (Figure 3H)	***P.hei* Tian & Chen, sp. n.**

### Pseudopraon hei

Taxon classificationAnimaliaHymenopteraBraconidae

Tian & Chen sp. n.

http://zoobank.org/9715BD3E-C3AA-445A-B478-0A1B36CAEBEF

Figure 3

#### Description.

Female. Body length 2.0 mm, fore wing length 1.8 mm.

**Figure 3.**
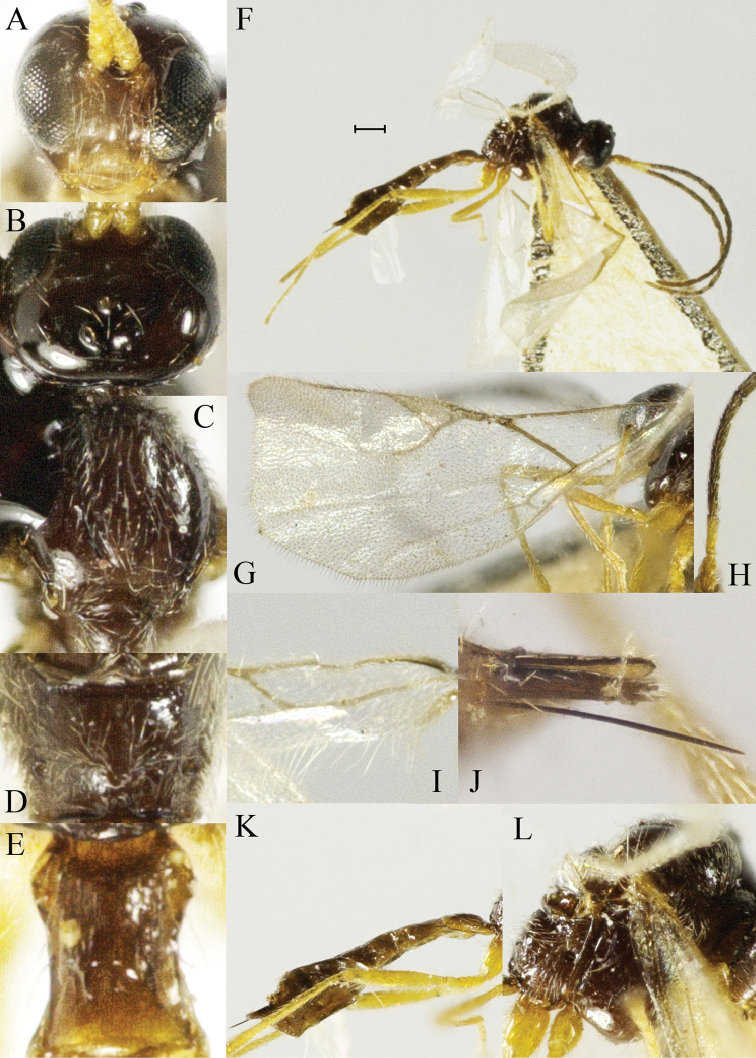
*Pseudopraonhei* Tian & Chen, sp. n. **A** head, anterior aspect **B** head, dorsal aspect **C** mesonotum, dorsal aspect **D** propodeum, dorsal aspect **E**T1, dorsal aspect **F** habitus, lateral aspect **G** fore wing **H** antennae **I** hind wing **J** ovipositor & ovipositor sheath, lateral aspect **K** metasoma, lateral aspect **L** mesosoma, lateral aspect. Scale bars: 0.2 mm.

*Head*. Head transverse in dorsal view, slightly wider than mesoscutum, smooth and shiny, with sparsely long setae (Figure 3B). Eyes medium-sized, oval, sparsely setose (Figure 3A). Temple in dorsal view 0.85 times as long as eye. Malar space equal to 0.2× longitudinal diameter of eye. POL 1.4× Od. Width of face 1.25× its height and 0.4× width of head. Face with dense setae. Clypeus oval, raised with several long setae, tentoriocular line equal to 0.25 of intertentorial line. Maxillary palp 4-segmented, labial palp 2-segmented (Figure 3A). Antenna filiform, with 18 segments. F1 approx. 1.2 times longer than F2. F1 4.0× as long as wide, F2 3.1× as long as wide (Figure 3H).

Mesosoma. Mesonotum with central and lateral lobes densely pubescent. Notauli deep and distinct throughout (Figure 3C). Propodeum glabrous, with dense long setae (Figure 3D).

*Wings*. Fore wing: apical margin with long setae, which are longer than setae on wing membrane. Pterostigma triangular, 3.4× as long as wide. Distal abscissa of 1-R1 (= metacarp) 0.7× as long as the length of pterostigma. Vein r+3-SR (= radial vein) slightly longer than the width of pterostigma, shorter than 1-R1 (5:7), do not reach the wing apex. 1-SR+M totally absent. Vein m-cu+2-M feebly pigmented, but distinctly present (Figure 3G). Hind wing with a complete cell, apical margin with long setae, which are longer than setae on wing membrane.

*Metasoma*. T1 nearly smooth, 1.2× longer than width at spiracle level, with long setae close to lateral corners. Ovipositor sheath with some long setae and its apex obtuse (Figure 3J).

*Colour*. Head dark brown, face somewhat paler, clypeus and mandibles yellowish to light brown. Maxillary and labial palpi white to yellowish. Antennal scape, pedicel and F1 yellowish. Remainder of antenna and mesoscutum dark brown. Wings hyaline with brown venation. Legs yellowish to light brown, apices of tarsi dark. Metasoma and ovipositor sheath dark brown to brown.

**Male.** Unknown.

#### Host.

Unknown.

#### Material examined.

**Holotype**: ♀, S China, Yunnan, Kunming, 30.III.1981, He Jun-Hua, No.811140.

#### Distribution.

China (Yunnan).

#### Taxonomic remarks.

This species is the second known species of this genus and can be easily differentiated from the type species, *P.mindariphagum* Starý, 1975 by having the flagellomere of antenna with more segments and the apex of ovipositor sheath sparsely setose (versus densely pubescent).

#### Etymology.

The new species is named in honour of Prof. Jun-Hua He (ZJUH), who also collected the holotype, for his valuable contribution to the taxonomy of parasitoid wasps in China.

## Supplementary Material

XML Treatment for Areopraon

XML Treatment for Areopraon chui

XML Treatment for Pseudopraon

XML Treatment for Pseudopraon hei
